# Cognitive constraints on motor imagery

**DOI:** 10.1007/s00426-015-0656-y

**Published:** 2015-03-11

**Authors:** Stephan F. Dahm, Martina Rieger

**Affiliations:** UMIT-University for Health Sciences Medical Informatics and Technology, Eduard Wallnöfer-Zentrum 1, 6060 Hall in Tyrol, Austria

## Abstract

Executed bimanual movements are prepared slower when moving to symbolically different than when moving to symbolically same targets and when targets are mapped to target locations in a left/right fashion than when they are mapped in an inner/outer fashion [Weigelt et al. (Psychol Res 71:238–447, 2007)]. We investigated whether these cognitive bimanual coordination constraints are observable in motor imagery. Participants performed fast bimanual reaching movements from start to target buttons. Symbolic target similarity and mapping were manipulated. Participants performed four action conditions: one execution and three imagination conditions. In the latter they indicated starting, ending, or starting and ending of the movement. We measured movement preparation (RT), movement execution (MT) and the combined duration of movement preparation and execution (RTMT). In all action conditions RTs and MTs were longer in movements towards different targets than in movements towards same targets. Further, RTMTs were longer when targets were mapped to target locations in a left/right fashion than when they were mapped in an inner/outer fashion, again in all action conditions. RTMTs in imagination and execution were similar, apart from the imagination condition in which participants indicated the start and the end of the movement. Here MTs, but not RTs, were longer than in the execution condition. In conclusion, cognitive coordination constraints are present in the motor imagery of fast (<1600 ms) bimanual movements. Further, alternations between inhibition and execution may prolong the duration of motor imagery.

## Introduction

Motor imagery designates the mental simulation of movements without actual body movements (Jeannerod, 1995). It is assumed to rely on similar processes as motor execution, i.e., imagination and execution are assumed to be functionally equivalent (functional equivalence hypothesis, e.g., Jeannerod, 1995). Nevertheless, sometimes differences between imagination and execution are observed (Decety, Jeannerod & Prablanc, 1989; Cerritelli, Maruff, Wilson & Currie, 2000). If the hypothesis of functional equivalence of imagination and execution holds, factors that constrain executed movements should also constrain imagined movements. To the best of our knowledge, in previous studies mainly the presence of biomechanical and motor constraints in imagery was investigated (Papaxanthis, Schieppati, Gentili & Pozzo, 2002; Papaxanthis, Pozzo, Kasprinski & Berthoz, 2003; Decety & Michel, 1989; Frak, Paulignan & Jeannerod, 2000), but rarely the presence of cognitive constraints. In the present study we therefore investigated the impact of cognitive bimanual coordination constraints on motor imagery.

The hypothesis of functional equivalence has been supported by studies using functional brain imaging, which show that similar brain areas are active during imagination and execution (Hanakawa, Honda, Okada, Fukuyama & Shibasaki, 2003; Lotze et al., 1999). Further support for the hypothesis of similar processes during imagination and execution comes from studies using mental chronometry. Studies using the mental chronometry paradigm investigate temporal similarities of imagined and executed movements (Jeannerod, 1994; Guillot & Collet, 2005). It is assumed that similar durations of execution and imagination (of the same movement) and positive correlations between those durations are caused by similar neural mechanisms. Similarities in durations of imagined and executed movements have been shown for a variety of different cyclical activities such as walking (Decety et al., 1989), rowing (Barr & Hall, 1992), speed skating (Oishi, Kasai & Maeshima, 2000), and an unfamiliar pedalo task (Munzert, 2002). Likewise, highly automated movements like writing (Decety & Michel, 1989), and reaching (Maruff & Velakoulis, 2000) show similar durations of imagination and execution. Correlations of the durations of imagination and execution, reflecting that individual differences between participants are preserved in imagery, have also been observed, for example in typing (Rieger, 2012) and in walking (Decety et al., 1989).

Several studies were concerned with the question whether biomechanical and motor constraints, which have an impact on movement execution, influence the duration of motor imagery. It has been shown that biomechanical and motor constraints, like inertial and gravitational constraints (Papaxanthis et al., 2002, 2003), Fitts’ Law (Cerritelli et al., 2000; Decety & Jeannerod, 1996; Lorey et al., 2010; Maruff et al., 1999; Radulescu, Adam, Fischer & Pratt, 2010), difficulty of a grasping movement (Frak et al., 2000), and performance differences between the left and the right hand (e.g., writing speed, Decety & Michael, 1989) influence imagination in a similar way as they influence execution. For instance, Cerritelli and colleagues (2000) investigated whether Fitts’ Law (Fitts, 1954) can be observed for imagined movements. Fitts’ Law states that movement difficulty, which is a function of movement amplitude and target width, determines movement duration (Fitts, 1954). Cerritelli and colleagues (2000) asked participants to imagine performing a pointing task to targets of different size. Imagined pointing durations conformed to Fitts’ Law. Altogether, the above-mentioned results support the hypothesis that biomechanical and motor constraints affect motor imagery.

However, other results indicate that not all biomechanical and motor constraints affect imagination in the same way as execution. For instance, compensatory muscle force, which people exert when executing movements with added weight, does not influence the durations of imagined movements (Decety et al., 1989; Cerritelli et al., 2000). Further, slower performance of the non-dominant hand in comparison to the dominant hand is more pronounced in imagined than in executed movements (Maruff et al., 1999). In contrast to the above-mentioned studies showing that gravitational constraints are taken into account during imagery (Papaxanthis et al., 2002, 2003), microgravity does not seem to be taken into account (Chabeauti, Assaiante & Vaugoyeau, 2012). Further, the imagination of movements in awkward and uncommon postures (Parsons, 1994), and unfamiliar movements, like typing in a different style than usually (Rieger, 2012) does not follow the same constraints as the execution of those movements. In addition, adequate movement duration during imagery depends on movement expertise (Reed, 2002). These results suggest that biomechanical and motor constraints sometimes do not influence imagination to the same degree as they influence execution.

In many tasks not only biomechanical and motor constraints have an impact on performance, but perceptual and cognitive constraints also play a role. For instance, in sequential finger tapping cognitive constraints due to chunking, and biomechanical constraints due to the anatomy of the fingers can be observed. Chunking influences timing of tapping movements, whereas the anatomy of the fingers influences movement trajectories (Loehr & Palmer, 2007). Cognitive and perceptual constraints seem to affect imagination. For instance, both imagination and execution are sensitive to an orientation illusion, created by a tilted background grating, when participants are asked to perform a posture selection task in which they either grasp a bar with the thumb on the left or right side (Glover, Dixon, Castiello & Rushworth, 2005). Further, when participants are asked to perform finger–thumb opposition movements to a metronome, neural differences (investigated using fMRI) between syncopated (peak flexion between metronome beats) and synchronized (peak flexion with metronome beats) coordination patterns persist in motor imagery. This reflects that imagination, like execution, is constrained by higher level cognitive processes, such as timing and planning (Oullier, Jantzen, Steinberg & Kelso, 2005).

In the present study, we investigated similarities of execution and imagination in a bimanual coordination task in which performance is mainly governed by cognitive constraints. In bimanual coordination movements are performed with both hands. Coordination performance strongly depends on whether the two hands do the same or different things (Swinnen & Wenderoth, 2004). Two patterns in particular have received a lot of attention in bimanual coordination research: symmetric coordination, in which the movement of the hands is mirrored along the body midline (e.g., both hands move to the body midline at the same time) and parallel coordination (as a specific case of asymmetric coordination) in which both hands move in the same direction in external space (e.g., both hands move to the left at the same time). Symmetric movements are easier to perform than asymmetric movements (e.g., Spijkers, Heuer, Kleinsorge & van der Loo, 1997). Performance of bimanual movements can be governed by several types of constraints (Swinnen & Wenderoth, 2004). Such constraints can be due to the structure of the motor system and biomechanics (Cardoso de Oliviera, 2002; Heuer, Kleinsorge, Spijkers & Steglich, 2004; Salter, Wishart, Lee & Simon, 2004, Swinnen, Dounskaia, Walter & Serrien, 1997). Because of neuronal cross-talk between the hemispheres which issue the motor commands for the two arms, similar movement parameters for both arms are beneficial during movement preparation due to interhemispheric interactions in the corpus callosum, and during movement execution due to efferent projections (Cardoso de Oliviera, 2002; Swinnen et al., 1997). Such cross-talk during movement programming affects imagination in a similar way as it affects execution (Heuer, Spijkers, Kleinsorge & van der Loo, 1998). Importantly, in recent years evidence has accumulated that perceptual (e.g., Mechsner, Kerzel, Knoblich & Prinz, 2001) and cognitive constraints (e.g., Diedrichsen, Hazeltine, Kennerly & Ivry, 2001; Diedrichsen, Ivry, Hazeltine, Kennerly & Cohen, 2003; Weigelt, Rieger, Mechsner & Prinz, 2007) also play an important role for bimanual coordination performance.

Weigelt et al. (2007) asked participants to perform bimanual tasks in which the targets for each hand were either located at same or different distances (Experiment 1) or in same or different directions (Experiment 2). In Experiment 2, two different symbolic target cues were mapped to four target locations (two for each hand) either in a left/right or inner/outer fashion. Depending on the mapping, a certain pair of target cues could either result in symmetric or parallel movements of the two arms. Thus, the paradigm provided a dissociation between the symbolic equivalence of target locations (same vs. different) and the physical similarity of movements (symmetric vs. parallel). Participants reacted and moved slower to different than same targets. Further, participants reacted slower when symbolic cues were mapped to targets in a left/right fashion compared to an inner/outer fashion. Motor constraints (i.e., whether movements were symmetric or parallel) had no effect on durations. Thus, performance was governed by cognitive constraints which depend on target similarity and whether the mapping of targets to locations in the environment is easy.

In the present study we used a task similar to Weigelt et al. (2007, Experiment 2). Participants were asked to perform bimanual movements towards two of four possible target locations, either in same or different directions. We mapped two different target cues to the four target locations either in a left/right or inner/outer fashion. Thus, as in Weigelt et al. (2007) movements in the same direction could be performed either to same or to different targets, depending on the mapping. The same holds for movements in different directions. In addition to Weigelt et al. (2007) we asked participants to either execute or imagine the bimanual movements. We expected that cognitive constraints of executed movements also affect motor imagery because the presence of movement constraints in motor imagery has been shown for a variety of tasks (Cerritelli et al., 2000; Decety & Michael, 1989; Frak et al., 2000; Papaxanthis et al., 2002). Consequently, movements to different targets should be slower than movements to same targets (cf. Diedrichsen et al., 2001, 2003; Weigelt et al., 2007) and movements in a left/right mapping should be slower than in an inner/outer mapping (cf. Weigelt et al., 2007), regardless of whether they are imagined or executed. If there are no biomechanical or motor constraints observable in execution (cf. Weigelt et al., 2007), such constraints should not be found in motor imagery.

We were further interested in whether target similarity and mapping constrain both movement preparation and movement execution. Movement preparation implies the specification of movement parameters, whereas movement execution implies the overt contraction of the muscles which are activated by the transmission of movement parameters to the limbs (Heuer et al., 1998). In order to measure movement preparation and movement execution separately, we set up three imagination conditions in which participants indicated movement initiation (IMA-start), termination of the movement (IMA-end), or both (IMA-start–end). We expected that cognitive constraints are reflected in both phases during imagination, at least to the degree they are apparent in execution (cf. Weigelt et al., 2007). The three different imagination conditions are also interesting for another reason: in all imagination conditions the movement is partly executed and partly imagined. It was of interest to investigate how alternations between imagined and executed movement elements may affect imagery durations. One may assume that alternations between imagination and execution may prolong durations, because imagery requires the inhibition of the movement (Jeannerod, 2001) which needs to be overcome when another part of the movement is executed. Most alternations occur in the IMA-start–end condition, less in the IMA-start and IMA-end, and none in the EXE condition. Durations might therefore be affected accordingly.

Apart from the possibility to investigate cognitive constraints in imagery, the task is interesting because it measures imagery on a shorter time scale than most other tasks used to investigate imagery, which usually last at least several seconds (for an overview see Guillot & Collet, 2005). Short imagery durations were so far mainly investigated using implicit imagery tasks. In implicit imagery tasks participants are not instructed to perform imagery, but rather to do another task. Participants usually implicitly use imagery in order to perform the task (e.g., de’Sperati & Stucchi, 2000; Frak et al., 2000; Parsons, 1987). In an explicit imagery task, in which participants are instructed to perform imagery, Guillot, Collet and Dittmar (2004) observed longer imagination than execution durations for short movements. Orliaguet and Coello (1998) proposed that whereas in longer movements imagination and execution share similar processing systems, in short movements, imagination and execution are processed differently. However, specific task demands also seem to be important for the temporal equivalence of short movements. Muesseler, Wuehr and Ziessler (2014) found that in easier conditions response times were shorter in imagination than in execution, but in more difficult conditions they were longer. We had thus no specific expectations about the temporal equivalence of imagination and execution.

In addition to performance, we investigated the strength of kinesthetic/tactile and visual representation of different movement elements during execution and imagination using subjective rating scales. This was of interest first, because even when imagination and execution durations are similar, the movement might still be represented in a slightly different way. Given the equivalence hypothesis, no differences between imagination and execution in the strength of kinesthetic/tactile and visual representations should be observed. However, previous results indicate that kinesthesis/touch and vision might be less strongly represented in imagination than in execution (Rieger & Massen, 2014). Second, this allowed us to control whether participants performed the task as instructed. For example, in the IMA-start conditions no measurements were taken after participants released the start buttons, but participants were still asked to imagine performing the movement and pressing the target buttons. Lower strength of representation in the IMA-start condition than in the other imagination conditions might indicate that participants did not perform the task as instructed.

## Methods

### Participants

Originally 24 students participated in the experiment, but one participant was excluded from analysis, because he did not perform the task according to instructions and reported a lack of concentration during the experiment. Mean age of the remaining 23 participants was 24.5 years (SD = 5.4 years). Seventeen were female, and nineteen were right-handed, one left-handed and three ambidextrous, as assessed by the Edinburgh Handedness Inventory (Oldfield, 1971). They were paid 9 Euros/h or received course credit for their participation. The experiment was approved by the local ethics committee and participants gave informed consent.

### Material and apparatus

**Fig. 1:**
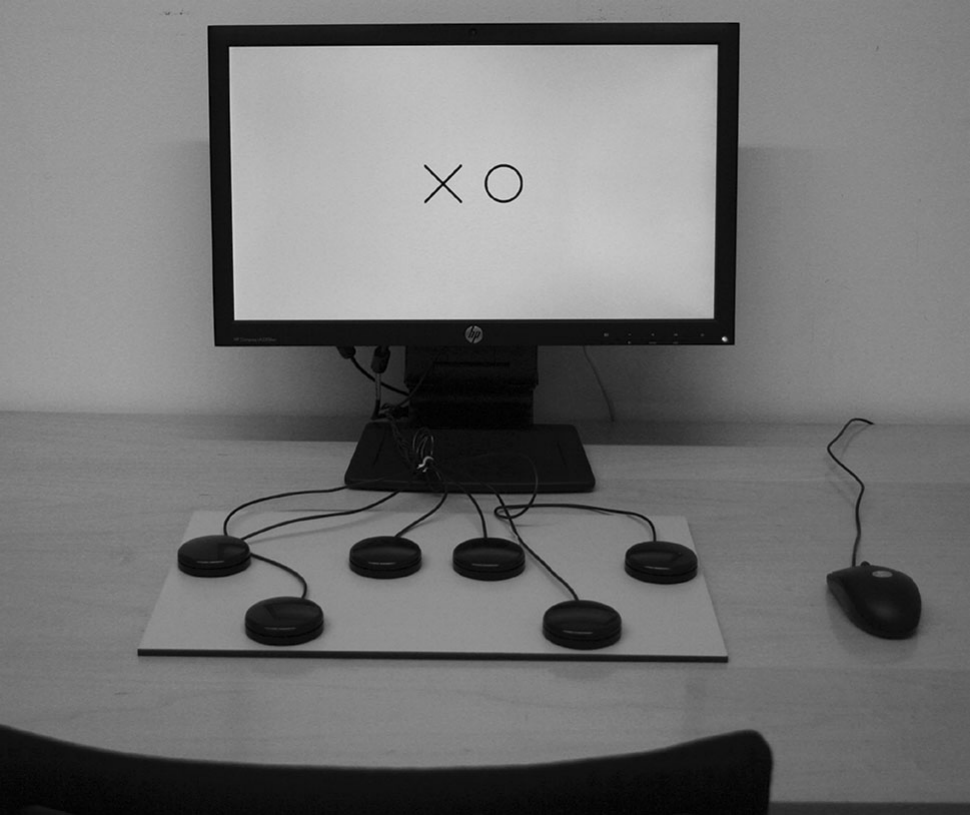
Experimental setup: stimuli on the screen and arrangement of the start and target buttons

A picture of the apparatus can be seen in Fig. 1. Stimuli were presented on an HPCompaqLA2206xc monitor (screen resolution 1920 × 1080 pixels, vertical refresh rate 76 Hz) located at a viewing distance of approximately 60 cm from participants. Stimuli consisted of circles and crosses (4.8 × 4.8 cm). Two stimuli were always presented concurrently in the center of the screen (distance between stimuli centers 6.8 cm). The left stimulus cued the movement of the left arm and the right stimulus cued the movement of the right arm. A go-back signal consisting of a blue square and a fixation cross (each 0.8 × 0.8 cm) were presented in the center of the screen. A board with six buttons (radius 6 cm) was placed at a distance of 20 cm from the edge of the table. The two buttons close to the participants (centers 24 cm from the edge of the table) were the start buttons (distance between centers of buttons: 12 cm). The other buttons were target buttons. They were located at an angle of 60° and at a distance of 15 cm in reference to the start buttons. The start buttons measured releases and presses, whereas the target buttons measured only presses. The experiment was programmed using the software Presentation (Version 16.3, http://www.neurobs.com).

Single questions were used to assess participants’ ease of imagery (from ‘very difficult’ to ‘very easy’), vividness of imagery (from ‘not vivid at all’ to ‘very vivid’), and concentration (from ‘very unconcentrated’ to ‘very concentrated’). In addition, participants were asked eight questions about their strength of representation of kinesthesis/touch (how it feels) and vision during the elements of the movement: being at the start buttons, releasing the start buttons, performing the movement, pressing the target buttons (from ‘not at all’ to ‘very clear’). Pilot data indicated that participants are not able to differentiate clearly between kinesthetic and tactile information in a similar context, thus we did not differentiate between them. An example of those questions is: ‘I felt/imagined to feel how my fingers pressed the start buttons’ (translated from German). Participants gave their ratings on a visual analog scale (15.9 cm), which was presented on the computer screen, by clicking with the computer mouse on the scale. The lowest score was defined as 0 and the highest score as 100.

### Procedure and design

**Fig. 2:**
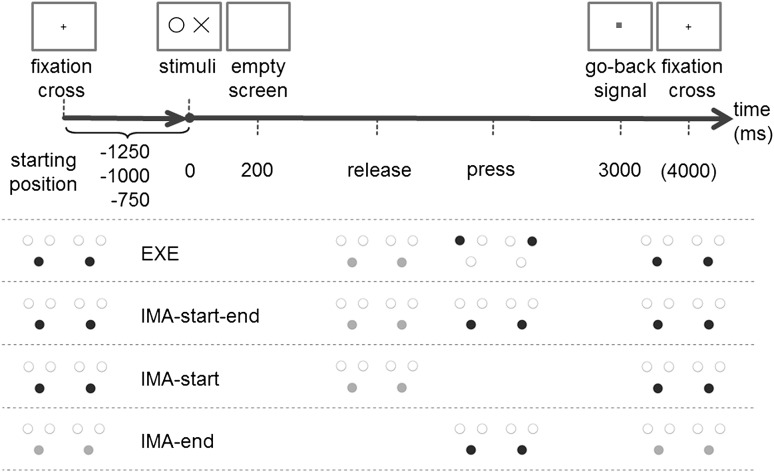
Trial procedure and timing for all action conditions. *White circles* indicate free buttons, *black circles* indicate button presses, and *grey circles* indicate button releases. After participants assumed the start position, a fixation cross was presented for 750, 1000 or 1250 ms. Then stimuli appeared for 200 ms on the screen (only one of four possible stimulus combinations is depicted). Participants then performed or imagined to perform the task depending on the action condition. After 3000 ms the go-back signal appeared and was presented until participants assumed the start position. Note that in IMA-end the start position was different from the other conditions. Participants kept their hands on the buttons without pressing them. The go-back signal was therefore presented for a fixed duration of 1000 ms

The procedure within a trial is depicted in Fig. 2. A trial started when participants pressed the two start buttons (start position), followed by the appearance of a fixation cross. The duration of the fixation cross randomly varied (750, 1000, or 1250 ms) to prevent anticipations. After that the stimuli were presented for 200 ms. In the execution condition (EXE) participants were asked to identify the correct target buttons, then release the start buttons, move to the target buttons and press them. A go-back signal was presented 3000 ms after the beginning of the stimuli. It disappeared as soon as participants assumed the start position.

In the three imagination conditions (IMA-start–end, IMA-start, IMA-end) arm movements towards the target buttons were not performed, but imagined. In the IMA-start–end condition participants were asked to indicate the beginning of the imagined arm movement by releasing the hands from the start buttons, to refrain from any further movement, and to press the start buttons when they arrived at the target buttons in their imagination. In the IMA-start condition participants indicated only the beginning of their imagined arm movement by releasing their hands from the start buttons. In the IMA-end condition the start position was different from the other conditions: at the beginning of a trial participants were asked to rest their hands on the start buttons, but not to press them. Participants were asked to press the start buttons as soon as they arrived at the target buttons in their imagination. Participants then released the start buttons, and were thus again in the start position. Therefore, the go-back signal had a fixed duration of 1000 ms. Participants were instructed to react as fast and accurately as possible in all conditions. During imagination participants were instructed to imagine how it feels to perform the movement. This was done in order to promote an internal imagery perspective and to reduce between participant variability in imagery styles. All instructions were presented in written form on the computer screen. An experimenter was present the whole time in order to make sure participants followed the instructions and to answer any questions.

**Fig. 3:**
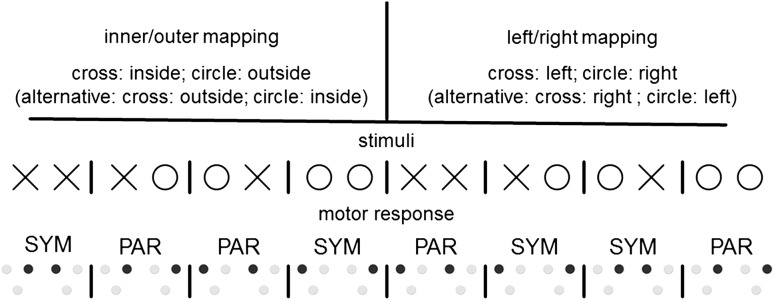
Schematic depictions of the mapping conditions. The task required motor execution and motor imagery to two of four target locations. *Circles* and *crosses* served as stimuli, specifying the target for each hand separately. Participants performed the task in two mapping conditions (*inner*/*outer* mapping and *left*/*right* mapping). In both mappings two different assignments of stimuli to target buttons are possible (only one of those is illustrated for each mapping). The assignments were counterbalanced between participants. *Black dots* represent the corresponding target buttons for the response. In each mapping, two stimulus combinations result in symmetric (SYM) and parallel (PAR) movements

The four action conditions were performed under two mapping conditions (see Fig. 3). In the inner/outer mapping same stimuli were associated with movements to the inside or outside. In the left/right mapping same stimuli were associated with movements to the left or right side. For instance, in the inner/outer mapping participants may have been asked to move to an inner target button if a cross is presented and an outer target button if a circle is presented. In the left/right mapping, participants may have been asked to move to a left target button if a cross is presented and to a right target button if a circle is presented. The specific mappings were counterbalanced across participants.

Each participant was assessed in two sessions. In the first session participants learned either the inner/outer mapping or the left/right mapping and performed 60 trials as practice. After a short break, another 20 trials were practiced. If they were not executed correctly, those 20 trials were repeated until participants performed all of them correctly (*M* = 1.2 repetitions in the inner/outer mapping and *M* = 1.8 repetitions in the left/right mapping). Following the learning of the mapping, participants performed the task in the four action conditions, which were presented blockwise. Each action condition started with 16 practice trials, which were followed by 80 experimental trials. Stimulus combinations were presented in randomized order, with the restriction that the same combination was presented maximally twice in a row. After each action condition, participants answered the questions about ease and vividness of imagery, concentration, and strength of representation. In the second session, approximately 1 week later, participants learned the other mapping. The procedure was the same as in the first session. The order of mapping conditions and the order of action conditions were counterbalanced across participants. The order of action conditions was the same in each session.

### Data analysis

A total of 3.4 % of the trials were not included in the analysis for the following reasons: (a) participants reacted before the stimuli appeared or within the first 200 ms after the stimuli appeared (anticipations), or (b) participants did not respond to the targets or responded only with one hand. Reaction time (RT) was defined as the median duration participants needed to release the start buttons, and movement time (MT) was defined as the median duration from the release of the start buttons to the press of the target buttons. In addition to RT and MT we calculated the total time of RT and MT (RTMT). All data were averaged over the left and right hand. Note that not all dependent variables are available in all action conditions. Movement errors (movements in which at least one hand terminated at the wrong target button) were included in the analysis because movement errors cannot be determined in the imagination conditions (*M* = 0.4 % in EXE). To prevent potential effects of accuracy, participants trained the task as long as they performed a series of 20 correct movements, as described above. Responses to ease and vividness of imagery, concentration, and strength of representation were averaged over the two mappings. Dependent variables were analyzed using repeated-measures analysis of variance (ANOVA). If Mauchly’s test indicated that the assumption of sphericity was violated, we report Huynh–Feldt corrected degrees of freedom and *p* values. Further comparisons were conducted using *t* tests with a Sidak-adjusted alpha or additional ANOVAs. Where appropriate we report minimum (*F*_min_, *p*_min_, *η*_*p*min_^2^) or maximum (*F*_max_, *p*_max_, *η*_*p*max_^2^) statistical values. Pearson correlations between imagination durations and execution durations were calculated separately for all combinations of the factors target and mapping. From those correlations we calculated the average correlation for RT, MT, and RTMT by using *z*-transformed values (Fisher’s *z* transformation). The correlations reported are reconverted from the average Fisher’s *z* values. Average correlations were compared using Fisher’s *z* test. Statistical significance was set at *p* < 0.05.

## Results

### Movement preparation: reaction time (RT)

**Fig. 4:**
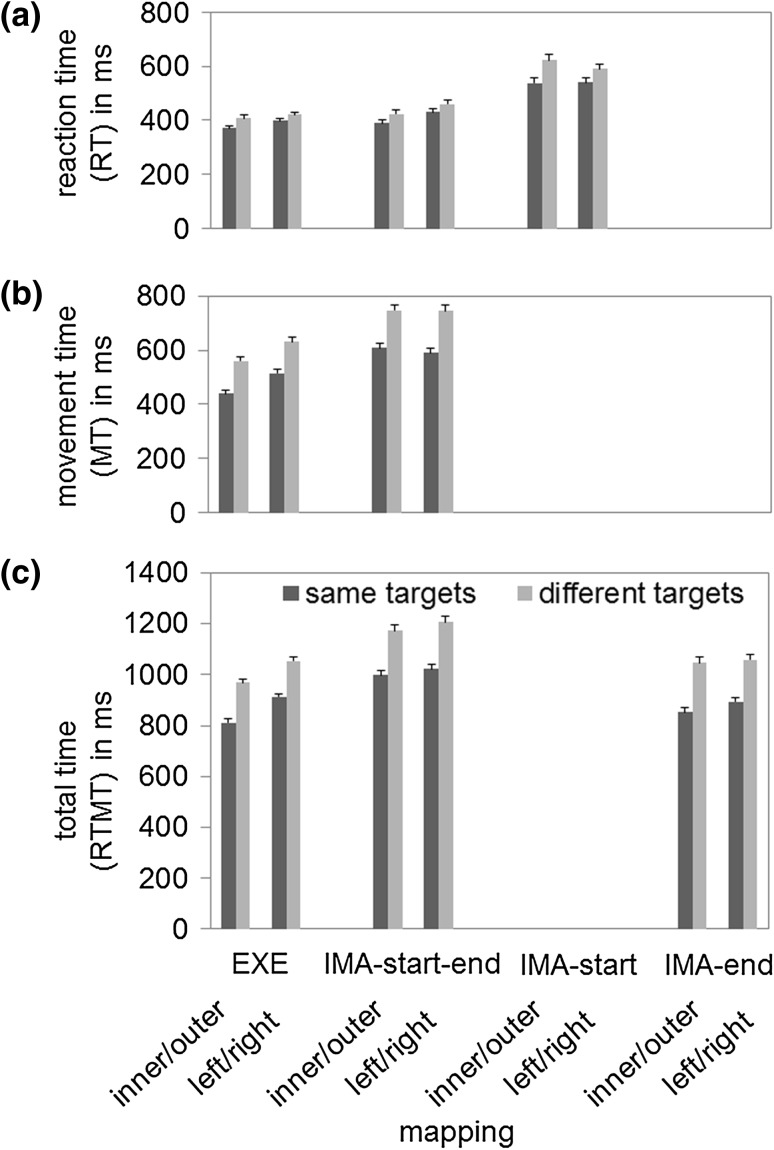
Mean reaction times (RT, **a**), movement times (MT, **b**), and combined RT and MT (RTMT, **c**), depending on target, mapping, and action. *Error bars* represent standard errors

Means and standard errors of RT can be seen in Fig. 4a. A repeated-measures ANOVA with the factors action (EXE, IMA-start–end, and IMA-start), target (same, different), and mapping (inner/outer, left/right) was performed on RT. A significant main effect of target, *F*(1, 22) = 13.11, *p* = 0.002, *η*_*p*_^2^ = 0.37, indicated that RT was longer with different (*M* = 487 ms) than with same targets (*M* = 445 ms). A significant main effect of action, *F*(2, 44) = 8.12, *p* = 0.001, *η*_*p*_^2^ = 0.27, indicated that RT was longer in IMA-start (*M* = 573 ms) than in EXE (*M* = 399 ms, *p* = 0.013) and IMA-start–end (*M* = 427 ms, *p* = 0.011). EXE and IMA-start–end did not differ significantly from each other (*p* = 0.86). All remaining effects were not significant [all *F* < 1 apart from target × action: *F*(1, 37) = 2.72, *p* = 0.09, *η*_*p*_^2^ = 0.11].

### Movement execution: movement time (MT)

Means and standard errors can be seen in Fig. 4b. A repeated-measures ANOVA with the factors action (EXE, IMA-start–end), target (same, different), and mapping (inner/outer, left/right) was performed on MT. A significant main effect of target, *F*(1, 22) = 26.33, *p* < 0.001, *η*_*p*_^2^ = 0.55, indicated that MT was longer with different (*M* = 667 ms) than with same targets (*M* = 536 ms). A significant main effect of action, *F*(1, 22) = 8.46, *p* = 0.008, *η*_*p*_^2^ = 0.28, indicated that MT was longer in IMA-start–end (*M* = 673 ms) than in EXE (*M* = 530 ms). A significant interaction between action and mapping, *F*(1, 22) = 7.28, *p* = 0.013, *η*_*p*_^2^ = 0.25, indicated that MT was longer in the left/right mapping than the inner/outer mapping in EXE (difference = −72 ms, *p* = 0.002), but not in IMA-start–end (difference =+10 ms, *p* = 0.739). All remaining effects were not significant [mapping: *F*(1, 22) = 3.32, *p* = 0.08, *η*_*p*_^2^ = 0.13; mapping × target: *F*(1, 22) < 1; target × action: *F*(1, 22) = 2.68, *p* = 0.12, *η*_*p*_^2^ = 0.11; target × mapping × action: *F*(1, 22) < 1].

### Movement preparation and movement execution (RTMT)

Means and standard errors can be seen in Fig. 4c. A repeated-measures ANOVA with the factors action (EXE, IMA-start–end, IMA-end), target (same, different), and mapping (inner/outer, left/right) was performed on RTMT. A significant main effect of target, *F*(1, 22) = 35.38, *p* < 0.001, *η*_*p*_^2^ = 0.62, indicated that RTMT was longer with different (*M* = 1081 ms) than with same targets (*M* = 913 ms). A significant main effect of mapping, *F*(1, 22) = 7.22, *p* = 0.013, *η*_*p*_^2^ = 0.25 indicated that RTMT was longer in the left/right mapping (M = 1024 ms) than the inner/outer mapping (*M* = 970 ms). A significant main effect of action, *F*(2, 44) = 5.2;7 *p* = 0.009, *η*_*p*_^2^ = 0.19 indicated that RTMT was longer in IMA-start–end (M = 1100 ms) than in EXE (*M* = 929 ms, *p* = 0.039) and IMA-end (*M* = 963 ms, *p* = 0.034). EXE and IMA-end did not differ significantly from each other (*p* = 0.90). All remaining effects were not significant [action × mapping: *F*(1, 44) = 2.74, *p* = 0.08, *η*_*p*_^2^ = 0.1; mapping × target: *F*(1, 22) < 1; target × action: *F*(1, 30) = 1.94, *p* = 0.17, *η*_*p*_^2^ = 0.08; target × mapping × action: *F*(1, 44) = 1.12, *p* = 0.34, *η*_*p*_^2^ = 0.05].

### Correlations between durations of imagination and execution

Correlations between durations of imagination and execution are shown in Table 1. Correlations ranged from *r* = 0.27 to *r* = 0.83 (critical *r* for a test against zero = 0.35). The average correlations of RT, MT, and RTMT did not significantly differ from each other (RT × MT: *Z* = 1.71, *p* = 0.09; RT × RTMT: *Z* = 1.78, *p* = 0.08; MT × RTMT: *Z* = 0.07, *p* = 0.94).

**Table 1:** Pearson correlations of executed (EXE) and imagined (IMA-start–end, IMA-start, IMA-end) movements depending on mapping and target condition, and averaged over those conditions, separately for reactions times (RT), movement times (MT) and total times (RTMT)

	Inner/outer mapping		Left/right mapping		Average
	Same targets	Different targets	Same targets	Different targets	
RT					
EXE × IMA-start–end	0.28	0.45*	0.47*	0.27	0.37*
EXE × IMA-start	0.37*	0.32	0.42*	0.35*	
MT					
EXE × IMA-start–end	0.60*	0.68*	0.75*	0.83*	0.73*
RTMT					
EXE × IMA-start–end	0.61*	0.72*	0.70*	0.73*	0.74*
EXE × IMA-end	0.70*	0.81*	0.74*	0.81*	

*RT* reaction time, *MT* movement time, *RTMT* total time, i.e., reaction time and movement time

* *p* < 0.05 (critical *r* = 0.35)

### Ease and vividness of imagery, concentration, and strength of representation

Means and standard errors of ease of imagery, vividness of imagery, and concentration can be seen in Table 2. Repeated-measures ANOVAs with the factor action (IMA-start–end, IMA-start, IMA-end) revealed no significant effect of action on ease of imagery, *F*(2, 44) < 1, and on vividness of imagery, *F*(2, 44) < 1. A repeated-measures ANOVA with the factor action (EXE, IMA-start–end, IMA-start, IMA-end) revealed no significant effect of action on concentration, *F*(3, 66) = 1.22, *p* = 0.31, *η*_*p*_^2^ = 0.05.

**Table 2:** Means and standard deviations (in parenthesis) of participants’ rating of the ease of imagery, vividness of imagery, and concentration during the task, separately for each action condition

	EXE	IMA-start–end	IMA-start	IMA-end
Ease of imagery	–	68 (16)	71 (17)	68 (16)
Vividness of imagery	–	69 (16)	66 (18)	66 (16)
Concentration during task	79 (14)	78 (11)	74 (16)	74 (12)

**Fig. 5:**
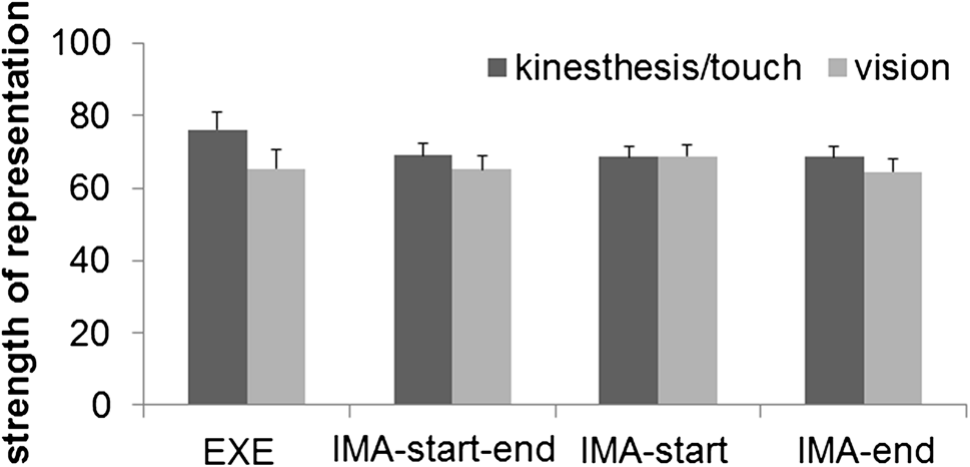
Mean strength of representation depending on action and modality. *Error bars* represent standard errors

Means and standard errors of strength of representation can be seen in Fig. 5. A repeated-measures ANOVA with the factors action (EXE, IMA-start–end, IMA-start, IMA-end), modality (kinesthesis/touch, vision) and movement element (button presses at start position, releasing start buttons, movement to target buttons, and pressing target buttons) was performed on strength of representation. A significant interaction between action and modality, *F*(3, 63) = 5.58, *p* = 0.002, *η*_*p*_^2^ = 0.21 was observed. All remaining effects were not significant [action: *F*(2, 36) = 1.22, *p* = 0.30, *η*_*p*_^2^ = 0.06; modality: *F*(1, 21) = 3.88, *p* = 0.06, *η*_*p*_^2^ = 0.16; movement element: *F*(3, 63) = 2.02, *p* = 0.12, *η*_*p*_^2^ = 0.09; action × movement element: *F*(5, 104) = 1.65, *p* = 0.16, *η*_*p*_^2^ = .07; modality × movement element: *F*(2, 48) = 1.75, *p* = 0.18, *η*_*p*_^2^ = 0.08; action × modality × movement element: *F*(6, 133) < 1].

For a more detailed analysis of the interaction between action and modality, we calculated repeated-measures ANOVAs with the factors modality and movement element for each action condition separately. A main effect of modality in EXE indicated that the strength of representation was higher for kinesthesis/touch than vision (difference = 11, *F*(1, 22) = 18.4, *p* < .001, *η*_*p*_^2^ = 0.46). No such effect was observed in any of the imagination conditions (IMA-start–end: difference = 4; IMA-end: difference = 0; IMA-end: difference = 4; *F*_max_(1, 22) = 1.4, *p*_min_ = 0.25, *η*_*p*max_^2^ = 0.06). Further, separate ANOVAs for each modality with the factors action and movement element were computed. The representation of kinesthesis/touch was stronger in EXE than in the imagination conditions (*F*_min_(1, 22) = 5.7, *p*_max_ = 0.026, *η*_*p*min_^2^ = 0.21), but the representation of vision did not significantly differ between action conditions (*F*_max_(1, 22) < 1).

## Discussion

The aim of the present study was to investigate the influence of cognitive constraints on motor imagery. For this aim we compared three imagination conditions (IMA-start–end, IMA-start, IMA-end) with overt execution (EXE) in a bimanual coordination task. Participants performed movements of both arms towards same or different targets, which were mapped to target locations in an inner/outer fashion or a left/right fashion. Movement preparation and movement execution were longer to different targets than to same targets. Total time was longer with the left/right mapping than with the inner/outer mapping. Movement preparation was longer in IMA-start than in EXE and movement execution was longer in IMA-start–end than in EXE. Correlations of imagined and executed movements were all positive. The strength of representation of vision did not significantly differ between action conditions, but kinesthesis/touch was represented stronger during execution than imagination.

We observed that movements to different targets were prepared and executed slower than movements to same targets. This is in accordance with previous results showing that target similarity affects movement preparation and movement execution (Weigelt et al., 2007). One may argue that the increased duration with different targets arises due to participants’ need to decode two different stimuli rather than two similar stimuli. However, in a similar task Weigelt et al. (2007) used stimulus masking (in order to ensure that participants start processing stimuli immediately) and response precuing (Rosenbaum, 1983). They showed that the target similarity effect was still present with a precuing interval of 500 ms. At this time both cues must have been fully processed. Thus, slower movements towards different targets than towards same targets reflect a cognitive constraint of bimanual coordination (Weigelt et al., 2007). As we observed such an effect in all action conditions, the data indicate that this cognitive constraint is present in motor imagery. We further observed longer total times with a left/right mapping than an inner/outer mapping in all action conditions. Better performance in a symmetrically arranged, easier environment represents another cognitive constraint of bimanual coordination (Weigelt et al., 2007), and our data indicate that this cognitive constraint is also present in motor imagery. Further, our data showed positive correlations between the durations of imagined and executed movements. This shows that participants, who were slower in execution than others, were also slower in imagination. This provides further evidence for a functional similarity of imagination and execution. The present results complement and extend previous findings showing that effects of neuronal cross-talk during programming of bimanual movements are apparent in motor imagery (Heuer et al., 1998). In addition, they go in line with neurological findings showing that stimulus and coordination constraints influence imagination and execution in a similar way (Oullier et al., 2005).

If task performance had been limited by biomechanical or motor constraints, slower parallel than symmetric movements should have been observed. Such an effect should have been apparent in an interaction between mapping and target, such that the difference between same and different targets is larger in the inner/outer mapping than in the left/right mapping. When participants are instructed with an inner/outer mapping, same targets coincide with symmetric movements and different targets coincide with parallel movements. When participants are instructed with a left/right mapping, same targets coincide with parallel movements and different targets coincide with symmetric movements. However, parallel movements were not significantly slower than symmetric movements, not even during execution. Thus, we conclude that performance in the present task was not limited by biomechanical or motor constraints. This is in line with previous findings using similar tasks (Diedrichsen et al., 2001, 2003; Kunde, Krauss & Weigelt, 2009; Weigelt et al., 2007).

We did not observe significantly longer durations with the left/right than the inner/outer mapping when we analyzed movement preparation and movement execution separately. However, such an effect was observed in total times. These findings partly diverge from the results of Weigelt et al. (2007), who observed longer durations in movement preparation (but not movement execution) with the left/right mapping than with the inner/outer mapping. One explanation for the present results might be that some participants initiated their movements before they had fully prepared it. The observation of a longer preparation phase in the IMA-start condition might be explained in a similar way: in all action conditions participants were instructed to start their movement only when they knew to which targets they should move. By this means we intended to separate movement preparation from movement execution. However, it might be that in EXE and IMA-start–end participants initiated their movements too early, whereas in IMA-start they initiated their movement after finishing movement preparation. IMA-start differs from the other action conditions, because no further timing was indicated by participants after movement initiation. This might have enforced participants to finish movement preparation completely, before they indicated movement initiation.

Several explanations are possible for the observation that movement execution was longer in IMA-start–end than in EXE. First, it might be that people attend more to the details of a movement during imagination than during execution. For instance, during imagination of a complex gymnastic vault participants report acoustic and kinesthetic representations, whereas those representations are not reported to the same degree during execution (Calmels, Holmes, Lopez & Naman, 2006). If movements are highly automated, attention to details may disrupt automatic processes and performance (Beilock, Carr, Mahon & Starkes, 2002; Logan & Crump, 2009). Correspondingly, attention to details during imagery is assumed to result in longer imagery than execution durations (Calmels et al., 2006). However, our results indicate that neither kinesthesis/touch nor vision is represented stronger during imagination than execution. Second, in IMA-start–end the actual hand positions when indicating target presses (at start buttons) were different from the imagined hand positions (at target buttons) and correspondingly the actual hand trajectories and the imagined hand trajectories also differed. This may have resulted in interference and thus longer durations. However, the same interference should have occurred in IMA-end, which did not take significantly longer than EXE, rendering this explanation unlikely. A third explanation, which most likely explains the present data, is that alternations between execution (indicating the start), inhibition (imagined movement), and again execution (indicating the end) in IMA-start–end might have made the task more difficult, resulting in longer durations in this condition. No inhibition was required in EXE, and only one alternation between inhibited (imagined start and movement) and executed (indicating the end) movement elements was necessary in IMA-end.

Interestingly, imagination durations of the whole task were only longer in IMA-start–end than EXE, but IMA-end and EXE did not significantly differ from each other. This challenges the assumption that imagination durations of short movements (<3 s) are always longer than execution durations (Grealy & Shearer, 2008). If temporal equivalence depends on task duration, we should have found longer imagination than execution durations in all imagination conditions, which was not the case. An explanation might be that rather than (or in addition to) the duration of a task, the characteristics of the movement are important even in relatively short movements. In the present study we used short and simple arm movements. In contrast, walking as investigated by Grealy and Shearer (2008) is relatively long and includes the whole body.

If the result of longer durations in IMA-start–end in comparison to the other action conditions is indeed due to alternations between inhibition and execution, this has important implications for the way imagination conditions are realized in future research using similar tasks. The IMA-start–end condition has the advantage that it is the only condition which offers a way to measure movement preparation and movement execution separately from each other. However, it has the disadvantage that imagination durations are more likely to be longer than execution durations compared to other imagination conditions. If one is not interested in having a separate measure for the execution phase (and one should bear in mind that movement preparation and movement execution are not always clearly separated), it might be advisable to measure the duration of imagery in a similar way as in the IMA-end condition. Vividness of imagery, ease of imagery, and the strength of representation of vision and kinesthesis/touch did not differ significantly between imagination conditions. Thus, none of the imagination conditions seems to be more difficult to imagine than the others.

We were further interested in the strength of kinesthetic/tactile and visual representations during imagination in comparison to execution. We therefore assessed the strength of representation of those two modalities after each action condition. Kinesthesis/touch was represented stronger than vision in execution, which was not the case in imagination. Correspondingly, kinesthesis/touch was more strongly represented in execution than in imagination. The present results are partly consistent with previous findings which indicated stronger visual and kinesthetic representations in execution than in imagination in a drawing task (Rieger & Massen, 2014). In the present study, the lower strength of representation of kinesthesis/touch in imagination than in execution might be caused by the absence of tactile and kinesthetic feedback from some movement elements during imagery. However, as tactile/kinesthetic feedback was not absent in all movement elements in the different imagination conditions, one could have expected that action conditions and movement elements interact with each other. This was not the case. It is possible that participants were not able to report their representations of each movement element separately. Rather, reports may have been influenced by the overall impression of representations during the whole movement. No significant difference between imagination and execution was found in the strength of visual representations. One explanation might be that in the present task visual representations do not depend on the movement itself, but rather on the visibility of the movement space. When participants perform reaching movements they do not watch their hands, but rather look at the target location before the movement is initiated (Helsen, Elliott, Starkes & Ricker, 2000). In the present study, it was not necessary to imagine this visual aspect of the task, as the target buttons were visible to participants during all action conditions. Given this, one may have expected a stronger representation of target presses than of other movement elements. Again, it might be that reports were influenced by the overall impression of representations during the whole movement. However, no differences in strength of kinesthetic/tactile and visual representations between movement elements might also indicate that participants complied with the instructions to imagine the whole movement in all imagination conditions. Compliance with the instructions is further supported by the ratings of vividness of imagery and ease of imagery, which did not differ significantly between imagination conditions, and concentration, which did not differ significantly between all action conditions.

We emphasized that in contrast to previous studies, which investigated biomechanical and motor constraints in imagery, we investigated cognitive constraints. One may, however, argue that constraints which are observed in imagery are always cognitive, as no overt movement takes place (e.g., Oullier et al., 2005). It is indeed not always easy to distinguish between motor and perceptual-cognitive constraints. Particularly, much debate exists concerning the contribution of the respective constraints in coordination performance (e.g., Oullier et al., 2005; Mechsner et al., 2001; Swinnen & Wenderoth, 2004). However, we do not think that the presence or absence of constraints in imagery is an adequate method to distinguish between motor-related or cognitive constraints. First, we think it is difficult to conceive how inertial and gravitational constraints (Papaxanthis et al., 2002, 2003) or performance differences between the left and the right hand (Decety & Michael, 1989), which are present in imagery, could be regarded as ‘cognitive’. Second, studies using functional brain imaging indicate activation of similar brain areas during imagination and execution, which extend to motor-related areas (e.g., Hanakawa et al., 2003; Lotze et al., 1999). Thus, we think that not only cognitive, but also motor constraints can be present in imagery. However, we admit that it is sometimes difficult to distinguish between cognitive and motor processes. But this applies not only to imagination, but also to execution.

The results of the present study have implications for mental practice, in which motor imagery is used in a systematic way in order to improve performance (Driskell, Copper & Moran, 1994). First, our results and previous results (Heuer et al., 1998; Oullier et al., 2005) suggest that different types of coordination constraints are present in imagery. Thus, it should be possible to effectively apply mental practice to activities which require a high amount of coordination between different limbs, like rowing. Second, our results suggest that functional equivalence of imagination and execution can occur in very short reactions to a stimulus. Thus, mental practice might be successfully applied to very short reactions, like starting to sprint when the start signal is given in a sprinting competition. Mental practice for movements of short durations has so far mainly been investigated using sequential movements similar to piano playing (e.g., Pascual-Leone et al., 1995), but to the best of our knowledge not for short reactions to stimuli. Third, alternations between inhibited and executed movement elements might be problematic in mental practice. Multiple alternations between inhibited and executed movement elements might weaken the effects of mental practice due to switching costs which disturb the flow of the movement. Fourth, when designing mental practice interventions, great care should be taken about the choice of modalities which are used for practice, because of task-dependent differences. For example, reaching movements as in the present study require a stronger representation of kinesthesis/touch, whereas other tasks like drawing (Rieger & Massen, 2014) require a stronger representation of vision.

## Conclusion

In conclusion, results confirm that slower movements to different than same targets represent the primary constraint of bimanual coordination in symbolically cued reaching movements (Diedrichsen et al., 2003; Weigelt et al., 2007). We were able to show that cognitive constraints (moving to same targets and moving in an ‘easy’ environment) of bimanual coordination are present in motor imagery. Even though movement durations were short (<1 s), mostly no significant differences between durations of imagination and execution were observed. The functional equivalence of imagination and execution is further reflected in positive correlations between durations of imagination and execution. This strengthens the hypothesis that motor imagery is based on similar processes as motor execution. However, alternations of executed and inhibited movement elements might prolong imagery durations. Further, not all aspects of a movement might be represented equally strongly in imagination and execution.
